# Toward Depth-Resolved Analysis of Plant Metabolites by Nanospray Desorption Electrospray Ionization Mass Spectrometry

**DOI:** 10.3390/molecules27217582

**Published:** 2022-11-04

**Authors:** Sangwon Cha, Gyouwoong Jun, Yougyeong Park, Sung Jun An, Donghoon Lee

**Affiliations:** 1 Department of Chemistry, Dongguk University, Seoul 04620, Korea; 2 Department of Chemistry, Hankuk University of Foreign Studies, Yongin 17035, Korea

**Keywords:** ambient desorption ionization, nanospray desorption electrospray ionization, mass spectrometry, plant, metabolite

## Abstract

Nanospray desorption electrospray ionization (nano-DESI) is one of the ambient desorption ionization methods for mass spectrometry (MS), and it utilizes a steady-state liquid junction formed between two microcapillaries to directly extract analytes from sample surfaces with minimal sample damage. In this study, we employed nano-DESI MS to perform a metabolite fingerprinting analysis directly from a *Hypericum* leaf surface. Moreover, we investigated whether changes in metabolite fingerprints with time can be related to metabolite distribution according to depth. From a raw *Hypericum* leaf, the mass spectral fingerprints of key metabolites, including flavonoids and prenylated phloroglucinols, were successfully obtained using ethanol as a nano-DESI solvent, and the changes in their intensities were observed with time via full mass scan experiments. In addition, the differential extraction patterns of the obtained mass spectral fingerprints were clearly visualized over time through selected ion monitoring and pseudo-selected reaction monitoring experiments. To examine the correlation between the time-dependent changes in the metabolite fingerprints and depth-wise metabolite distribution, we performed a nano-DESI MS analysis against leaves whose surface layers were removed multiple times by forming polymeric gum Arabic films on their surfaces, followed by detaching. The preliminary results showed that the changes in the metabolite fingerprints according to the number of peelings showed a similar pattern with those obtained from the raw leaves over time.

## 1. Introduction

Ambient desorption ionization (ADI) methods for mass spectrometry (MS) have been extensively developed since the invention of desorption electrospray ionization (DESI) [1]. The key feature of ADI is that it enables direct sampling and the ionization of analytes directly from an object to be analyzed with minimal sample pretreatment. Therefore, ADI MS has been extensively used in obtaining chemical fingerprints directly from various solid objects, including pharmaceuticals, food materials, soils, industrial goods, and even human skin [2]. Among various ADI methods, nanospray-DESI (nano-DESI), which was invented by Laskin group in 2010, utilizes liquid extraction-based analyte desorption and electrospray ionization [3]. Nano-DESI MS has been applied to the in situ analysis of various classes of compounds, including drugs, lipids, petrochemicals, proteins, microbial metabolites, and organic aerosol compounds, and also to the study of electrochemical reactions [3,4,5,6,7,8,9].

In nano-DESI, two microcapillaries (i.e., primary and secondary nanospray) are closely configured at a certain angle to form a steady-state liquid junction between them. Then, a charged solvent is delivered through the primary capillary at a flow rate of a few hundred nanoliters per minute, and analytes are extracted at the liquid-junction–sample-surface interface [3,10]. This analyte extraction procedure induces minimal sample damage and can be achieved at a high spatial resolution (~10 μm) [11,12]. These extraction characteristics distinguish nano-DESI from DESI, which induces a rather forceful analyte extraction by directing a pneumatically assisted charged solvent spray to sample surfaces [1,13]. The analytes extracted at the liquid junction in nano-DESI are then instantly transferred through a self-aspirating secondary capillary for nanoelectrospray ionization and MS analysis. Based on the fact that the minimally destructive extraction of analytes on outermost sample surfaces can be realized and that the extracted analytes are immediately ionized and detected, it can be hypothesized that the change in the nano-DESI mass spectral fingerprints over time can be related to the depth-wise distribution of analytes in a sample. To test this hypothesis, using nano-DESI MS, our group recently performed a chemical fingerprint analysis on inks in handwritten documents, and the distribution of ink chemicals according to depth was successfully probed through real-time chemical fingerprint changes obtained by nano-DESI MS [14].

Recently, as mass spectrometric imaging (MSI) techniques have been actively developed, it has become possible to directly acquire spatial distribution information of chemicals from various raw samples such as animal and plant tissues [15,16], microbial colonies [17], and foods [18]. Although most of MSI research focused on obtaining two-dimensional (2D) distribution information of chemicals, three-dimensional (3D) MSI studies have also been actively performed [19,20]. In 3D MSI, depth-wise molecular information of a sample is usually obtained in two ways. One way is to use sputtering- or laser ablation-based depth profiling and the other way is to analyze consecutive sections to obtain 2D images and reconstruct them in 3D [19]. However, 3D MSI research with plant tissue samples was very limited due to following characteristics of plant tissues. First, it is very hard to obtain consecutive tissue sections from plant samples, especially from thin organs such as flower petals and leaves. Second, soft and high water-containing plant tissues are easily distorted and shrunk when they are analyzed with vacuum MSI platforms. Depth profiling using atmospheric pressure laser ablation electrospray ionization MS [21,22] has been almost the only way for performing depth-dependent molecular analysis of intact plant tissue samples.

In this study, we investigated whether the depth-dependent analysis capability of nano-DESI MS could be applied to the challenging task of obtaining depth-wise chemical information from the plant tissues. We tried to acquire plant metabolite signals directly from a given location on a raw *Hypericum* leaf sample for a relatively long period of time (~15 min) and then investigated whether time-dependent differential extraction could be observed. Based on the obtained preliminary results, we could observe significantly different time-dependent extraction patterns between key plant metabolites, flavonoids, and prenylated phloroglucinols, via nano-DESI MS. In addition, we performed a nano-DESI MS analysis on surface-layer-removed leaf samples to examine the relationship between the time-dependent changes in metabolite fingerprints and depth-wise metabolite distributions. Then, we could find that the changes in the fingerprints of plant metabolites according to the number of peelings and the time-dependent changes in the obtained fingerprints from raw leaves using nano-DESI MS were similar. Overall, although depth resolution or chemical distribution information at a specific depth cannot be provided by the current methodology, we could prove that the differential distribution of metabolites according to depth can be probed directly from a plant leaf through nano-DESI MS.

## 2. Results and Discussion

### 2.1. Metabolite Fingerprinting of a Hypericum Raw Leaf Surface by Nano-DESI MS

**Figure 1:**
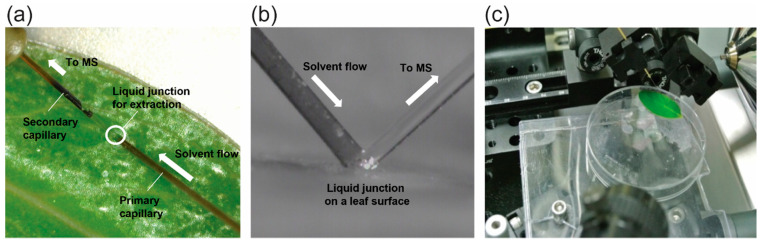
Nano-DESI MS of a *Hypericum* leaf. A liquid junction of a nano-DESI was in contact with a leaf surface, as seen in the (**a**) top view and (**b**) side view of the nano-DESI probe on the leaf. (**c**) Photo of the homebuilt nano-DESI MS interface for the chemical fingerprinting of plant leaf samples.

Metabolite fingerprinting experiments were performed on *Hypericum* raw leaves by nano-DESI MS (Figure 1). A raw leaf attached to an insulating plastic surface was placed under the nano-DESI probe, and the liquid junction formed between the two microcapillaries of the nano-DESI probe was positioned until it gently touched the leaf surface, as shown in Figure 1b. During the nano-DESI MS acquisition, the temporal changes in the metabolite fingerprints were recorded by keeping the liquid junction in contact with the leaf surface for a long period of time (>10 min). To realize steady and stable data acquisition for a long period of time at a given sample location, various nano-DESI solvents were tested. In the initial experiments, solutions containing chloroform (CHCl_3_) were employed, as CHCl_3_ is known to be an effective solvent in terms of removing and extracting wax chemicals (e.g., very long chain fatty acids and their derivatives), which are present on the outermost surfaces of plant leaves [23,24]. However, when CHCl_3_ was added to the spraying solvent, the liquid junction was not stable enough for nano-DESI MS to perform long-term data acquisition at one location. This is probably because dense CHCl_3_ is not viscous enough to retain the shape of the liquid junction. Therefore, we extensively tested non-CHCl_3_-containing solvents, and ethanol (EtOH) showed the best performance in terms of the long-term stability of the liquid junction. However, EtOH showed lower extraction efficiency against plant materials than CHCl_3_-containing solvents, and thus longer data acquisition was generally required.

**Figure 2:**
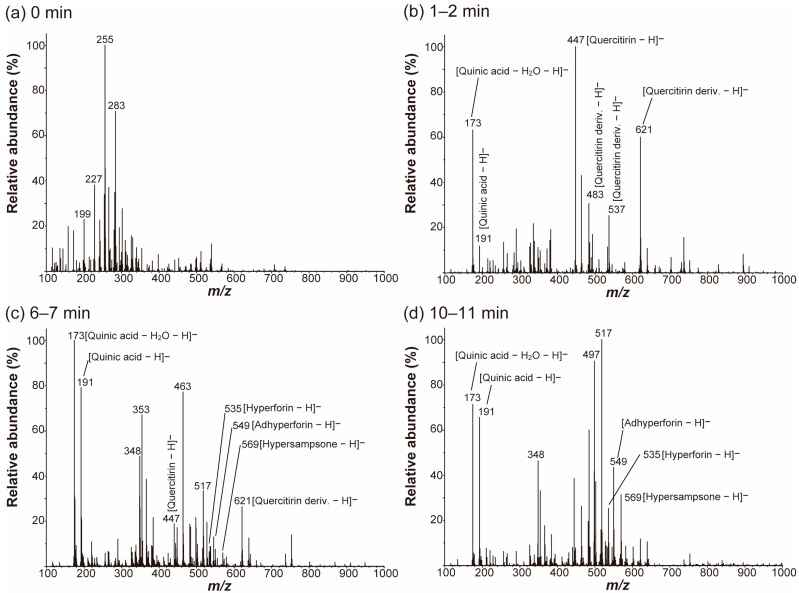
Time-segmented and averaged mass spectra of a *Hypericum* leaf obtained by nano-DESI MS in the negative ion mode. (**a**) 0 min, (**b**) 1–2 min; (**c**) 6–7 min; (**d**) 10–11 min. The spraying solvent was EtOH. The abbreviation “deriv.” denotes “derivative”.

**Figure 3:**
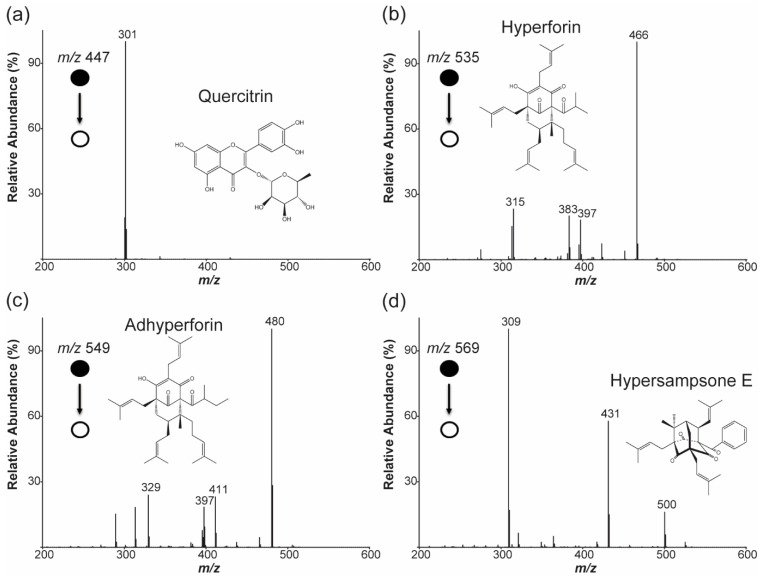
First-generation product ion spectra (MS/MS) for the ions detected in the spectrum in Figure 2 at (**a**) *m*/*z* 447, (**b**) *m*/*z* 535, (**c**) *m*/*z* 549, and (**d**) *m*/*z* 569. The tentatively identified molecule and its structure are indicated in each spectrum. Tentative identification was performed by comparing the observed product ions for a given precursor ion with the ions reported in the literature on the metabolite analysis of *Hypericum* species [25,26].

The time-segmented metabolite fingerprints directly collected from a raw *Hypericum* leaf surface in the negative ion mode are shown in Figure 2. At the beginning of acquisition (0 min), there were no signals except for those from the spraying solvents (Figure 2a). After ~1 min, ion signals from the plant metabolites appeared (Figure 2b–d). A tandem MS (MS/MS) analysis was performed on the major peaks detected from the leaf surface by nano-DESI MS, and some of these peaks were tentatively identified by comparing their product ion spectra shown in Figure 3 with previously reported ion spectra [25,26]. In the first 1–2 min, the deprotonated ions of quinic acid (*m*/*z* 173 and 191) and flavonoids, including quercitrin (*m*/*z* 447) and their derivatives (*m*/*z* 483, 537, and 621), were mainly observed (Figure 2b). After 5 min, prenylated phloroglucinol derivatives, such as hyperforin (*m*/*z* 535), adhyperforin (*m*/*z* 549), and hypersampsone (*m*/*z* 569), started to be detected, and both quercitrins and hyperforins appeared in the spectra after 6–7 min (Figure 2c). After ~10 min, in contrast, the deprotonated ions of phloroglucinols were dominant, and the signals of quercitrin and its derivatives were significantly decreased, as shown in Figure 2d.

Quinic acid is a cyclohexanecarboxylic acid found in many medicinal plants including *Hypericum* and has been known to exhibit antioxidant and antibacterial activities [27]. Quinic acid is the key component of chlorogenic acids, one of the most abundant phenolic acids in the plant kingdom [28]. Quercitrin (quercetin-*O*-rhamnoside) is a flavonoid found in many vegetables and *Hypericum* species [29], and it exhibits various bioactive properties, such as cytotoxicity, antioxidative activity, anti-inflammatory activity, and antiallergic activity [30,31,32]. Moreover, quercitrin has been known to inhibit the cell death or apoptosis induced by ultraviolet (UV) light [33,34]. Hyperforin and adhyperforin are derivatives of polycyclic polyprenylated acylphloroglucinols and are abundant in *Hypericum* species [35]. Hyperforins exhibit antidepressant activity, anti-inflammatory activity, and antitumoral effects [36,37,38,39]; however, they are susceptible to degradation under direct light exposure [40,41].

### 2.2. Selected Ion Monitoring and Pseudo-Selected Reaction Monitoring for the Major Ions

**Figure 4:**
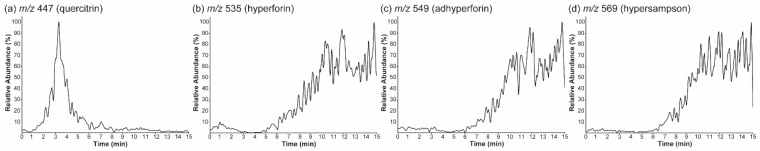
Selected ion monitoring (SIM) chronograms for the ions at (**a**) *m*/*z* 447, (**b**) *m*/*z* 535, (**c**) *m*/*z* 549, and (**d**) *m*/*z* 569, which were detected from a *Hypericum* leaf surface by nano-DESI MS. SIM experiments were performed with a mass window of ±1 Da.

To clearly visualize the temporal changes in the metabolite fingerprints obtained by nano-DESI MS, selected ion monitoring (SIM) experiments were performed for the representative ions: quercitrin at *m*/*z* 447, hyperforin at *m*/*z* 535, adhyperforin at *m*/*z* 549, and hypersampsone at *m*/*z* 569. The SIM chronograms of these ions were demonstrated, as shown in Figure 4. The SIM results clearly provided similar patterns to those collected in the full mass scan mode, as shown in Figure 2. The ions corresponding to quercitrin at *m*/*z* 447 started increasing after ~1 min and were dominant until 5 min (Figure 4a), and the ions corresponding to the prenylated phloroglucinol derivatives at *m*/*z* 535, 549, and 569 started increasing after 5 min and were dominant until the end of acquisition (Figure 4b–d). However, the SIM chronograms of the prenylated phloroglucinol derivatives also showed low signal levels during the first 5 min. These low signal levels could be from prenylated phloroglucinol derivatives; however, they could also be from other metabolites or background signals.

**Figure 5:**
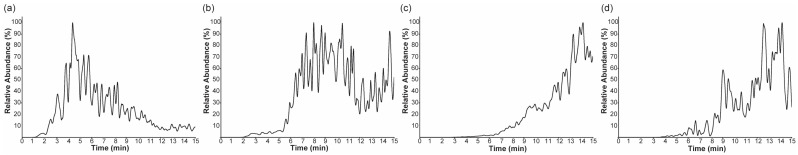
Psuedo-selected reaction monitoring (pSRM) chronograms for the transitions (**a**) *m*/*z* 447 → *m*/*z* 301, (**b**) *m*/*z* 535 → *m*/*z* 466, (**c**) *m*/*z* 549 → *m*/*z* 480, and (**d**) *m*/*z* 569 → *m*/*z* 309 obtained directly from a *Hypericum* leaf surface by nano-DESI MS. The normalized collision energy values were set to 30% for (**a**–**c**) and 35% for (**d**).

To acquire more selective time-dependent profiles for these metabolites, pseudo-selected reaction monitoring (pSRM) experiments were performed with transitions from the given precursor ions to major product ions found in the full MS/MS spectra (Figure 3): *m*/*z* 447 → *m*/*z* 301, *m*/*z* 535 → *m*/*z* 466, *m*/*z* 549 → *m*/*z* 480, and *m*/*z* 569 → *m*/*z* 309. As shown in Figure 5b–d, the pSRM chronograms of the prenylated phloroglucinol derivatives confirmed that the low level of the signals observed in their SIM chronograms (Figure 4) resulted from other metabolite or background signals and not from these metabolites.

### 2.3. Nano-DESI MS of the Surface-Layer-Removed Hypericum Leaves

**Figure 6:**
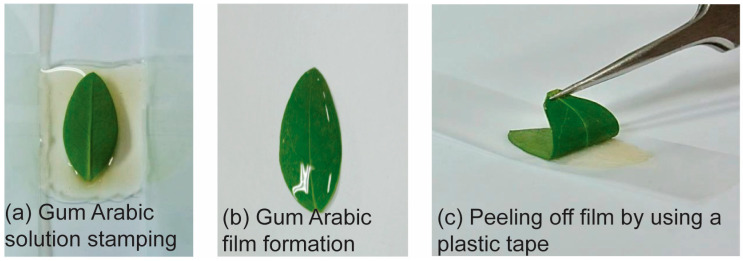
Mechanical peeling of a surface layer of a leaf by utilizing polymeric gum Arabic films. See Section 3 for the detailed procedure.

The time-dependent changes in the metabolic fingerprints obtained directly from leaves by nano-DESI MS showed that there is a clear difference among the metabolites in terms of extraction degree. However, this fact alone is not sufficient to conclude that the difference in the extraction degree is due to the difference in the metabolite distribution according to depth. To partially fulfill this gap, we tried to perform surface layer removal from a *Hypericum* leaf by applying a polymeric gum Arabic film to a leaf surface, followed by detaching (Figure 6) [42]. Through this mechanical peeling method, we could generally remove surface layers up to five to six times without any changes in the overall leaf shape and without tearing. However, the extent to which the leaf surface was peeled through this method was difficult to predict because the surface properties vary based on the number of times the surface is peeled off. In addition, the horizontal dislocation of surface chemicals and the contamination during the gum Arabic film formation could be problematic. Nevertheless, we adopted this method because we thought that it can be one of the few methods of exposing the inner layers of leaves with minimal impact on the vertical distribution of chemicals.

**Figure 7:**
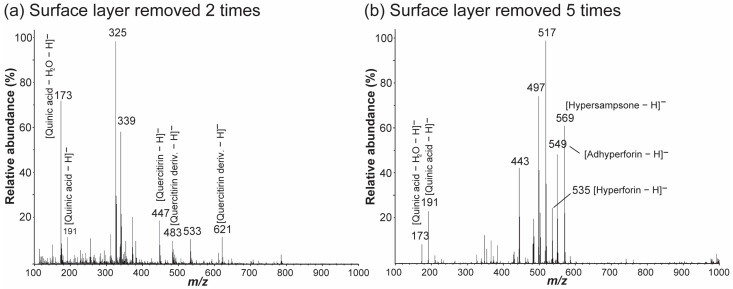
Nano-DESI mass spectra of the surface-layer-removed leaf samples in the negative ion mode. Mass spectra were acquired from the leaf surfaces whose surface layers were removed (**a**) two and (**b**) five times by polymeric gum Arabic film formation and detachment. The spraying solvent was EtOH. The abbreviation “deriv.” denotes “derivative”.

The metabolite fingerprints obtained from the surface-layer-removed leaves by nano-DESI MS are shown in Figure 7. Metabolite fingerprints from leaf samples of which surface layer were removed two and five times (Figure 7a,b) were similar to those extracted at different time windows from time-dependent metabolite fingerprinting data (Figure 2b,d) except two unknown signals at *m*/*z* 325 and at *m*/*z* 339 in Figure 7a. In other words, quercitrin and its derivatives were mainly detected in the leaf from which the surface layer was removed twice (Figure 7a), whereas hyperforins were mainly observed in the leaf from which the surface layer was removed 5 times (Figure 7b). However, it should be noted that the number of peelings required to observe the changing trend was different for each leaf sample. These results suggest that the time-course changes in the metabolite fingerprints collected from a raw leaf sample by nano-DESI MS can reflect the depth-wise distributions of plant metabolites.

## 3. Materials and Methods

### 3.1. Nano-DESI MS

#### 3.1.1. Nano-DESI Probe

A homebuilt nano-DESI probe was used, and it is composed of two fused silica microcapillaries with 150 μm OD and 50 μm ID (Polymicro Technologies LLC, Phoenix, AZ, USA), as shown in Figure 1a,b. The primary capillary (Figure 1b, left) was connected to a solution feeding capillary (360 μm OD and 100 μm ID fused silica) and to a platinum wire (Sigma-Aldrich, St. Louis, MO, USA) for electrical contact by utilizing a Micro-TEE connector (IDEX Heath & Science LLC, Oak Harbor, WA, USA). One end of the secondary capillary (Figure 1b, right) was in contact with the primary capillary at about 90°, and the other end of this capillary was a nanoelectrospray emitter with less than 10 μm ID. As shown in Figure 1b, the polyimide coating of the secondary capillary was removed to obtain a more stable and smaller liquid junction between the two capillaries. The nano-DESI solvent was fed by a syringe pump (Fusion 100 Touch, Chemyx, Inc., Stafford, TX, USA) at a flowrate of 300–600 nL/min. Various nano-DESI solvents were tested, including methanol (MeOH)/water (H_2_O)/CHCl_3_ (5:1:0.1, *v*/*v*), MeOH/H_2_O (5:1, *v*/*v*), EtOH, and EtOH/CHCl_3_ (5:1, *v*/*v*), and EtOH was finally selected to record the nano-DESI mass spectral profiles against the plant samples. All the used solvents except for deionized water were purchased from Fisher Scientific (Fairlawn, NJ, USA).

#### 3.1.2. Mass Spectrometry

A nano-DESI source was interfaced with a Thermo Finnigan LTQ XL linear ion trap mass spectrometer (Mountain View, CA, USA), as shown in Figure 1c. The lateral and vertical positions of the liquid junction were adjusted by the XYZ-positioning stage (Newport, San Jose, CA, USA) and monitored by two video cameras. Mass spectra were recorded in the negative ion mode using the full scan mode, SIM mode, or pSRM mode. The spray and capillary voltages were set to −5 kV and −10 V, respectively. For the pSRM mode acquisition, product ions were generated via collision-induced dissociation at a normalized collision energy of 30% or 35%. Mass spectral raw data were first processed by Thermo Xcalibur 2.2 (Thermo Fisher Scientific, Waltham, MA, USA) to generate mass spectra and ion chronograms. The generated mass spectra were further processed by mMass version 5.5 (http://www.mmass.org, accedded on 20 September 2022) for data presentation.

### 3.2. Plant Sample Preparation for Nano-DESI MS

*Hypericum patulum* “Hidcote” was obtained from a local botanical garden, and leaf samples were collected after ~12 weeks of growth. The leaf samples were used either without any further treatment (raw leaf) or after surface layer removal (surface-layer-removed leaf). Mechanical peeling of the leaf surface layers was performed using a previously reported method but with minor modifications [42]. Briefly, gum Arabic powder was first purified with CHCl_3_ by Soxhlet extraction. Then, an aqueous solution of gum Arabic (90% *w*/*w*) was prepared and applied onto a glass slide (~0.1 mL/cm^2^). Then, a leaf surface was gently stamped onto this solution (Figure 6a). After drying under ambient conditions, a polymeric gum Arabic film was formed on the leaf surface (Figure 6b). This polymeric film was peeled off using scotch tape (3M, St. Paul, MN, USA) (Figure 6c). For the nano-DESI MS analysis, a raw or surface-layer-removed leaf was attached to an insulating petri dish surface using a regular double-sided tape (3M, St. Paul, MN, USA).

## 4. Conclusions

In this study, we analyzed the metabolites directly from the *Hypericum* leaf surface by nano-DESI MS and investigated whether time-course extraction profiles obtained by nano-DESI MS could be related with plant metabolite distributions according to depth. Our results showed that well-known *Hypericum* metabolites, quercitrins and hyperforins were differentially extracted and detected by nano-DESI MS. Correlation of differential extraction profiles and depth-wise distributions of these metabolites were further confirmed by analyzing leaves whose surface layers were mechanically removed multiple times. Considering the properties of quercitrins that inhibit apoptosis by UV light [33,34] and the properties of hyperforins that are easily photo-degraded [40,41], it seems reasonable that quercitrins may be more abundantly distributed on the outermost surface than hyperforins, as shown in our results. However, this fact should be further confirmed by other methodologies. Overall, this study suggests the potential for nano-DESI MS to be applied to depth-dependent chemical analysis even for complex raw samples such as plants.

## Data Availability

The data presented in this study are available on request from the corresponding author.

## References

[1] Takáts Z., Wiseman J.M., Gologan B., Cooks R.G. (2004). Mass Spectrometry Sampling Under Ambient Conditions with Desorption Electrospray Ionization. Science.

[2] Shrestha S.A., Cha S. (2021). Ambient desorption/ionization mass spectrometry for direct solid material analysis. TrAC Trends Anal. Chem..

[3] Roach P.J., Laskin J., Laskin A. (2010). Nanospray desorption electrospray ionization: An ambient method for liquid-extraction surface sampling in mass spectrometry. Analyst.

[4] Eckert P.A., Roach P.J., Laskin A., Laskin J. (2012). Chemical Characterization of Crude Petroleum Using Nanospray Desorption Electrospray Ionization Coupled with High-Resolution Mass Spectrometry. Anal. Chem..

[5] Liu P., Lanekoff I.T., Laskin J., Dewald H.D., Chen H. (2012). Study of Electrochemical Reactions Using Nanospray Desorption Electrospray Ionization Mass Spectrometry. Anal. Chem..

[6] Watrous J., Roach P., Alexandrov T., Heath B.S., Yang J.Y., Kersten R.D., van der Voort M., Pogliano K., Gross H., Raaijmakers J.M. (2012). Mass spectral molecular networking of living microbial colonies. Proc. Natl. Acad. Sci. USA.

[7] Lanekoff I., Geydebrekht O., Pinchuk G.E., Konopka A.E., Laskin J. (2013). Spatially resolved analysis of glycolipids and metabolites in living Synechococcus sp. PCC 7002 using nanospray desorption electrospray ionization. Analyst.

[8] Hsu C.-C., Chou P.-T., Zare R.N. (2015). Imaging of Proteins in Tissue Samples Using Nanospray Desorption Electrospray Ionization Mass Spectrometry. Anal. Chem..

[9] Lanekoff I., Burnum-Johnson K., Thomas M., Cha J., Dey S.K., Yang P., Conaway M.C.P., Laskin J. (2015). Three-dimensional imaging of lipids and metabolites in tissues by nanospray desorption electrospray ionization mass spectrometry. Anal. Bioanal. Chem..

[10] Roach P.J., Laskin J., Laskin A. (2010). Molecular Characterization of Organic Aerosols Using Nanospray-Desorption/Electrospray Ionization-Mass Spectrometry. Anal. Chem..

[11] Lanekoff I., Heath B.S., Liyu A., Thomas M., Carson J.P., Laskin J. (2012). Automated Platform for High-Resolution Tissue Imaging Using Nanospray Desorption Electrospray Ionization Mass Spectrometry. Anal. Chem..

[12] Nguyen S.N., Sontag R.L., Carson J.P., Corley R.A., Ansong C., Laskin J. (2018). Towards High-Resolution Tissue Imaging Using Nanospray Desorption Electrospray Ionization Mass Spectrometry Coupled to Shear Force Microscopy. J. Am. Soc. Mass Spectrom..

[13] Venter A., Sojka P.E., Cooks R.G. (2006). Droplet Dynamics and Ionization Mechanisms in Desorption Electrospray Ionization Mass Spectrometry. Anal. Chem..

[14] Lee G., Cha S. (2021). Depth-Dependent Chemical Analysis of Handwriting by Nanospray Desorption Electrospray Ionization Mass Spectrometry. J. Am. Soc. Mass Spectrom..

[15] Buchberger A.R., Delaney K., Johnson J., Jillian J. (2018). Mass Spectrometry Imaging: A Review of Emerging Advancements and Future Insights. Anal. Chem..

[16] Boughton B.A., Thinagaran D., Sarabia D., Bacic A., Roessner U. (2016). Mass spectrometry imaging for plant biology: A review. Phytochem. Rev..

[17] Dunham S.J.B., Ellis J.F., Li B., Sweedler J.V. (2017). Mass Spectrometry Imaging of Complex Microbial Communities. Acc. Chem. Res..

[18] Yoshimura Y., Zaima N., Yukihiro Y. (2020). Application of Mass Spectrometry Imaging for Visualizing Food Components. Foods.

[19] Vos D.R.N., Ellis S.R., Balluff B., Heeren R.M.A. (2021). Experimental and Data Analysis Considerations for Three-Dimensional Mass Spectrometry Imaging in Biomedical Research. Mol. Imaging Biol..

[20] Seeley E., Caprioli R.M. (2012). 3D Imaging by Mass Spectrometry: A New Frontier. Anal. Chem..

[21] Nemes P., Barton A.A., Vertes A. (2009). Three-Dimensional Imaging of Metabolites in Tissues under Ambient Conditions by Laser Ablation Electrospray Ionization Mass Spectrometry. Anal. Chem..

[22] Nemes P., Barton A.A., Li Y., Vertes A. (2008). Ambient Molecular Imaging and Depth Profiling of Live Tissue by Infrared Laser Ablation Electrospray Ionization Mass Spectrometry. Anal. Chem..

[23] Millar A.A., Clemens S., Zachgo S., Giblin E.M., Taylor D.C., Kunst L. (1999). *CUT1*, an Arabidopsis Gene Required for Cuticular Wax Biosynthesis and Pollen Fertility, Encodes a Very-Long-Chain Fatty Acid Condensing Enzyme. Plant Cell.

[24] Razeq F.M., Kosma D.K., Rowland O., Molina I. (2014). Extracellular lipids of Camelina sativa: Characterization of chloroform-extractable waxes from aerial and subterranean surfaces. Phytochemistry.

[25] Hvattum E., Ekeberg D. (2003). Study of the collision-induced radical cleavage of flavonoid glycosides using negative electrospray ionization tandem quadrupole mass spectrometry. J. Mass Spectrom..

[26] Porzel A., Farag M.A., Mülbradt J., Wessjohann L.A. (2014). Metabolite profiling and fingerprinting of Hypericum species: A comparison of MS and NMR metabolomics. Metabolomics.

[27] Benali T., Bakrim S., Ghchime R., Benkhaira N., El Omari N., Balahbib A., Taha D., Zengin G., Hasan M.M., Bibi S. (2022). Pharmacological insights into the multifaceted biological properties of quinic acid. Biotechnol. Genet. Eng. Rev..

[28] Franklin G., Dias A. (2011). Chlorogenic acid participates in the regulation of shoot, root and root hair development in *Hypericum perforatum*. Plant Physiol. Biochem..

[29] Huang H.-S., Liaw E.-T. (2017). Extraction Optimization of Flavonoids from Hypericum formosanum and Matrix Metalloproteinase-1 Inhibitory Activity. Molecules.

[30] Camuesco D., Comalada M., Rodriguez-Cabezas M.E., Nieto A., Lorente M.D., Concha A., Zarzuelo A., Gálvez J. (2004). The intestinal anti-inflammatory effect of quercitrin is associated with an inhibition in iNOS expression. Br. J. Pharmacol..

[31] Choi S.-J., Tai B.H., Cuong N.M., Kim Y.-H., Jang H.-D. (2012). Antioxidative and anti-inflammatory effect of quercetin and its glycosides isolated from mampat (*Cratoxylum formosum*). Food Sci. Biotechnol..

[32] Truong V.-L., Ko S.-Y., Jun M., Jeong W.-S. (2016). Quercitrin from Toona sinensis (Juss.) M.Roem. Attenuates Acetaminophen-Induced Acute Liver Toxicity in HepG2 Cells and Mice through Induction of Antioxidant Machinery and Inhibition of Inflammation. Nutrients.

[33] Yang H.-M., Ham Y.-M., Yoon W.-J., Roh S.W., Jeon Y.-J., Oda T., Kang S.-M., Kang M.-C., Kim E.-A., Kim D. (2012). Quercitrin protects against ultraviolet B-induced cell death in vitro and in an in vivo zebrafish model. J. Photochem. Photobiol. B Biol..

[34] Yin Y., Li W., Son Y.-O., Sun L., Lu J., Kim D., Wang X., Yao H., Wang L., Pratheeshkumar P. (2013). Quercitrin protects skin from UVB-induced oxidative damage. Toxicol. Appl. Pharmacol..

[35] Müller W.E. (2003). Current St. John’s wort research from mode of action to clinical efficacy. Pharmacol. Res..

[36] Schempp C.M., Pelz K., Wittmer A., Schöpf E., Simon J.C. (1999). Antibacterial activity of hyperforin from St John’s wort, against multiresistant Staphylococcus aureus and gram-positive bacteria. Lancet.

[37] Schempp C.M., Kirkin V., Simon-Haarhaus B., Kersten A., Kiss J., Termeer C.C., Gilb B., Kaufmann T., Borner C., Sleeman J.P. (2002). Inhibition of tumour cell growth by hyperforin, a novel anticancer drug from St. John’s wort that acts by induction of apoptosis. Oncogene.

[38] Medina M.A., Martínez-Poveda B., Amores-Sánchez M.I., Quesada A.R. (2006). Hyperforin: More than an antidepressant bioactive compound?. Life Sci..

[39] Zanoli P. (2004). Role of Hyperforin in the Pharmacological Activities of St. John’s Wort. CNS Drug Rev..

[40] Brechner M.L., Albright L.D., Weston L. (2011). Effects of UV-B on Secondary Metabolites of St. John’s Wort (*Hypericum perforatum* L.) Grown in Controlled Environments. Photochem. Photobiol..

[41] Rizzo P., Altschmied L., Ravindran B.M., Rutten T., D’Auria J.C. (2020). The biochemical and genetic basis for the biosynthesis of bioactive compounds in *Hypericum perforatum* L., one of the largest medicinal crops in europe. Genes.

[42] Jetter R., Schäffer S. (2001). Chemical Composition of the *Prunus laurocerasus* Leaf Surface. Dynamic Changes of the Epicuticular Wax Film during Leaf Development. Plant Physiol..

